# Identification, characterization, and comparative genomic distribution of the HERV-K (HML-2) group of human endogenous retroviruses

**DOI:** 10.1186/1742-4690-8-90

**Published:** 2011-11-08

**Authors:** Ravi P Subramanian, Julia H Wildschutte, Crystal Russo, John M Coffin

**Affiliations:** 1 Department of Molecular Biology and Microbiology, Tufts University School of Medicine, Boston, MA 02111, USA

**Keywords:** evolution, endogenous retroviruses, human genome

## Abstract

**Background:**

Integration of retroviral DNA into a germ cell may lead to a provirus that is transmitted vertically to that host's offspring as an endogenous retrovirus (ERV). In humans, ERVs (HERVs) comprise about 8% of the genome, the vast majority of which are truncated and/or highly mutated and no longer encode functional genes. The most recently active retroviruses that integrated into the human germ line are members of the *Betaretrovirus*-like HERV-K (HML-2) group, many of which contain intact open reading frames (ORFs) in some or all genes, sometimes encoding functional proteins that are expressed in various tissues. Interestingly, this expression is upregulated in many tumors ranging from breast and ovarian tissues to lymphomas and melanomas, as well as schizophrenia, rheumatoid arthritis, and other disorders.

**Results:**

No study to date has characterized all HML-2 elements in the genome, an essential step towards determining a possible functional role of HML-2 expression in disease. We present here the most comprehensive and accurate catalog of all full-length and partial HML-2 proviruses, as well as solo LTR elements, within the published human genome to date. Furthermore, we provide evidence for preferential maintenance of proviruses and solo LTR elements on gene-rich chromosomes of the human genome and in proximity to gene regions.

**Conclusions:**

Our analysis has found and corrected several errors in the annotation of HML-2 elements in the human genome, including mislabeling of a newly identified group called HML-11. HML-elements have been implicated in a wide array of diseases, and characterization of these elements will play a fundamental role to understand the relationship between endogenous retrovirus expression and disease.

## Background

During the retrovirus infection cycle, viral genomic RNA is reverse transcribed into a DNA copy that is permanently integrated into the genomic DNA of the host. The integration of retroviral cDNA into the DNA of a germ cell occasionally results in an endogenous retrovirus (ERV), a provirus that is transmitted vertically to that host's offspring, and which may become fixed in the host species over time [1]. ERVs have been detected thus far in every animal species tested, including humans. ERVs contribute to approximately 8% of the human genome [2,3], and the vast majority of human ERVs (HERVs) lack infectious capacity due to accumulated nonsense mutations, insertions, and deletions of internal coding regions and/or long terminal repeats (LTRs). Despite the accumulation of deleterious mutations, a number of HERVs, most corresponding to relatively recent germline infections, have intact open reading frames (ORFs) that encode functional proteins and, in some cases, can form retrovirus-like particles [4-9].

The HERV-K clade of betaretrovirus-like endogenous retroviruses contains ten groups (HML-1-10) that are most closely related to mouse mammary tumor virus (MMTV), a causative agent for breast cancer in mice [10]. The most recently active retroviruses belong to the HML-2 group, which has been previously estimated to comprise roughly 60 proviruses and more than 2500 solitary LTRs (solo LTRs), resulting from intra- or inter-LTR recombination events [11,12]. HML-2 proviruses are further classified by the presence (type 1) or absence (type 2) of a 292 bp deletion at the *pol*-*env *junction [13], or based upon their LTR sequence [14]. Unique among HERVs, the HML-2 group includes human-specific proviruses, of which 11 are known to be insertionally polymorphic within the population [8,15-17]. The HML-2 insertion rate appears to have been approximately constant since the *Homo*-*Pan *divergence, suggesting that replication-competent HML-2 viruses may yet exist within the human population [16,18]. Despite the presence of replication-competent ERVs in other vertebrates, no infectious HERV has been observed to date. However, two engineered HML-2 proviruses, corresponding to the inferred common ancestor of the human specific elements, are weakly infectious [19,20].

Expression of HML-2 proviruses is known to be up-regulated in tissues associated with several diseases, including breast cancers [21-25], germ cell tumors [26-29], melanomas [30-33], ovarian cancer [34,35], leukemias/lymphomas [36,37], schizophrenia [38-41], and rheumatoid arthritis [42-45], as well as during HIV infection [46,47], in which transcripts, proteins, and even retrovirus-like particles originating from HML-2 proviruses have been observed. However, any functional consequences of this expression remain unknown. In general, research directed toward identifying individually expressed loci is limited, and knowledge of the specific loci being transcribed, as well as the reason(s) for their activation, are largely nonexistent. Just one study has investigated expression of individual proviruses, providing evidence for the differential transcription of > 20 HML-2 proviruses in normal and tumor-derived human tissues [28]. Although these approaches have provided information on the expression patterns of some HML-2 proviruses, a caveat is that polymorphic HML-2 proviruses may be missed, as features that differentiate these particular loci, such as sequence polymorphisms, remain uncharacterized. A more complete and up-to-date catalog of HML-2 elements will help to alleviate such difficulties.

Since its initial publication in 2001 [3], the human genome sequence has evolved through several builds. These have provided a powerful means of identifying and cataloging endogenous proviruses. However, changes in defined genome coordinates from one build to the next present a problem in confirming the genomic positions of specific sequences like HERVs, and have complicated the use of existing literature for the verification of individual HML-2 loci. Also, the fact that some members of the HML-2 group are polymorphic has led to their incomplete representation in the existing genome sequence. For example, the K103 provirus (located at chromosomal position 10p12.1) is represented as a solo LTR in all genome builds; however, the provirus has been sequenced and is publicly available in the NCBI nucleotide database [15]. Also missing from the published sequence is HERV-K113, located at 19p12 and arguably the most studied HML-2 provirus [8,48]. Furthermore, additional polymorphic HML-2 proviruses are likely to be identified with continued improvements in sequencing technologies and increased genome sequence information. Their characterization, in terms of integration site, structure and function, and in association to disease, will require a systematic catalog of described proviruses as a reference point for future analyses.

Here, we report a comprehensive analysis of HML-2 elements present within human DNAs. Through iterative data searching of the most recent human genome assembly (Feb. 2009 GRCh37/hg19), we have identified and characterized 91 proviruses, and 944 solo LTRs belonging to the HML-2 family. We have accounted for all known polymorphic HML-2 proviruses, including 10p12.1 (K103) and 19p12b (K113). We have also sequenced and included two previously uncharacterized HML-2 proviruses: one at chromosomal position 12q13.2 represented in the published genome as a solo LTR [16], and K105 [15], also published as a solo LTR and located within an unassembled genomic region. Finally, we have identified putative open reading frames (ORFs) for proviruses and determined the age of provirus and solo LTR elements. Together, these data provide the most up-to-date catalog of HML-2 elements within the human genome and represent, to our knowledge, the most complete and accurate dataset of its kind.

## Results

### Generation of a comprehensive HML-2 dataset

We mined the most recent human genome assembly (GRCh37/hg19) for sequences with strong similarity to HERV-K113 provirus using the BLAST-like alignment tool (BLAT) within the UCSC Genome Browser website [49]. K113 is the most recent germ-line integration known, with an allele frequency of ~16% and estimated to have formed ~1 million years ago [8,50]. Though the K113 provirus is not present in the published human genome, we were able to identify it at chromosome band 19p12 using GenBank sequences that contained flanking sequences (accession numbers: AF387849 and AF387847). By searching for proviruses with the highest percent identity to K113, we were able to identify 62 full-length or near full-length proviruses with > 87% nucleotide identity to the full-length K113 genome (Table 1). Our nomenclature for proviruses is consistent with Hughes and Coffin [51], where identification is based upon chromosome band location in the human genome. In the case of multiple proviruses in the same chromosome band, we labeled each provirus with an "a", "b", "c" etc. depending upon its order within the band. To this list, an additional four polymorphic proviruses were added: K113 itself, K103 (at 10p12.1), 12q13.2, and K105 (located within the unassembled centromeric region Un_g1000219) for an initial set of 66 proviruses. K103, K105, and 12q13.2 are represented in the current (and all previous) genome builds as solo LTRs. Full-length sequences for the K103 and K113 proviruses are available through GenBank (AF164611 and AY037928 respectively). The sequence of the K105 provirus was not included in the original report due to its location within highly repetitive DNA [15]; however, by examining the NCBI database we identified a provirus, "K111", that most likely represents human K105 (see Materials and Methods). Finally, the 12q13.2 provirus was one of few polymorphic proviruses to be identified since publication of the human genome [16], although its sequence was not deposited in the NCBI database. We have analyzed this provirus and provide its sequence for the first time here.

**Table 1:** Full-length and near full-length HML-2 proviruses

Locus	Alias	Chr	Orientation	Start	End	Oldest Common Ancestora	Estimated Age^f^	Polymorphic Alleles	ORFs	References
1p36.21b	K(OLDAL023753), K6, K76	**1**	(+)	13458305	13467826	Orangutan	22.47-40.68		*gag*	Reus et al., 2001

1p31.1	K4, K116, ERVK- 1	**1**	(+)	75842771	75849143	Human Specific	< 2	Provirus	*gag*	Hughes and Coffin, 2001

1q21.3		**1**	(-)	150605284	150608361	Orangutan	N/A			This Study

1q22	K102, K(C1b), K50a, ERVK-7	**1**	(-)	155596457	155605636	Human Specific	< 2			Barbulescu et al., 1999

1q23.3	K110, K18, K(C1a), ERVK-18	**1**	(+)	160660575	160669806	Gorilla^b^	7.81-14.14			Barbulescu et al., 199

1q24.1	K12	**1**	(-)	166574603	166580258	Rhesus	14.17-25.65			Romano et al., 2006

1q32.2		**1**	(-)	207808457	207812636	Orangutan	N/A			This Study

2q21.1		**2**	(-)	130719538	130722209	Human Specific	N/A			This Study

3p25.3	K11, ERVK-2	**3**	(-)	9889346	9896236	Orangutan	12.13-21.96*			Hughes and Coffin, 2001

3p12.3		**3**	(+)	75600465	75609150	Chimpanzee	N/A			This Study

3q12.3	K(II), ERVK-5	**3**	(+)	101410737	101419859	Gorilla^b^	5.51-9.98			Sugimoto et al., 2001

3q13.2	K106, K(C3), K68, ERVK-3	**3**	(-)	112743479	112752282	Human Specific	< 2	Provirus, soloLTR	*gag*	Barbulescu et al., 1999

3q21.2	K(I), ERVK-4	**3**	(+)	125609302	125618416	Human Specific	4.8-8.69			Sugimoto et al., 2001

3q24	ERVK-13	**3**	(-)	148281477	148285396	Human Specific	N/A		*gag*	This Study

3q27.2	K50b, K117, ERVK-11	**3**	(-)	185280336	185289515	Human Specific	< 2		*gag, pol*	Hughes and Coffin, 2001

4p16.3a		**4**	(+)	234989	239459	Rhesus	N/A			This Study

4p16.1a	K17b	**4**	(+)	9123515	9133075	Chimpanzee	16.84-30.49			Romano et al., 2006

4q13.2		**4**	(+)	69463709	69469223	Orangutan	17.09-30.95		*env*	This Study

4q32.1		**4**	(+)	161579938	161582360	Chimpanzee	N/A			This Study

4q32.3	K5, ERVK-12	**4**	(+)	165916840	165924068	Orangutan	9.24-16.73			Hughes and Coffin, 2001

4q35.2		**4**	(-)	191027414	191034701	Human Specific	13.07-23.67			This Study

5p13.3	K104, K50d	**5**	(-)	30487114	30496205	Human Specific	6.32-11.44			Barbulescu et al., 1999

5p12		**5**	(-)	46000159	46010002	Orangutan	13.39-24.24			This Study

5q33.3	K107/K10, K(C5), ERVK-10	**5**	(-)	156084717	156093896	Human Specific	< 2		*gag, pol*	Ono et al., 1986

6p22.1	K(OLDAL121932), K69, K20	**6**	(+)	28650367	28660735	Orangutan	19.55-35.4			Reus et al., 2001

6p21.1	K(OLDAL035587), KOLD35587	**6**	(-)	42861409	42871367	Rhesus	9.83-17.81			Reus et al., 2001

6q14.1	K109, K(C6), ERVK-9	**6**	(-)	78427019	78436083	Human Specific	< 2	Provirus, soloLTR	*gag, env*	Barbulescu et al., 1999

6q25.1		**6**	(+)	151180749	151183574	Rhesus	N/A		*env*	This Study

7p22.1a	K108L, K (HML.2-HOM), K(C7), ERVK-6	**7**	(-)	4622057	4631528	Human Specific	< 2	Provirus, Tandem Repeat, SoloLTR	*pol, env*	Barbulescu et al., 1999

7p22.1b	K108R, ERVK-6	**7**	(-)	4630561	4640031	Human Specific	< 2	Provirus, Tandem Repeat, SoloLTR	*pol, env*	Barbulescu et al., 1999

7q22.2	ERVK-14	**7**	(-)	104388369	104393266	Human Specific	N/A		*gag*	This Study

7q34	K(OLDAC004979), ERVK-15	**7**	(-)	141450926	141455903	Orangutan	N/A		*gag*	Reus et al., 2001

8p23.1a	K115, ERVK-8	**8**	(-)	7355397	7364859	Human Specific	4.87-8.82*	Provirus	*gag, pol, env*	Turner et al., 2001

8p23.1b	K27	**8**	(+)	8054700	8055725	Chimpanzee	16.09-29.13			Hughes and Coffin, 2001

8p23.1c		**8**	(-)	12073970	12083497	Chimpanzee	15-27.17			Hughes and Coffin, 2001

8p23.1d	KOLD130352	**8**	(-)	12316492	12326007	Human Specific^e^	15.22-27.56			Hughes and Coffin, 2001

8q11.1	K70, K43	**8**	(-)	47175650	47183661	Unknown^d^	23.46-42.48			Romano et al., 2006

8q24.3a		**8**	(-)	140472149	140475236	Human Specific	N/A			This Study

9q34.11	K31	**9**	(+)	131612515	131619736	Orangutan	12.43-22.51			Hughes and Coffin, 2001

9q34.3	K30	**9**	(-)	139674766	139684228	Orangutan	15.46-28			Hughes and Coffin, 2001

10p14	K(C11a), K33, ERVK-16	**10**	(-)	6867109	6874635	Gorilla^b^	7.36-13.32			Costas et al., 2001

10p12.1	K103, K(C10)	**10**	(+)	27182399	27183380	Human Specific	1.61-2.91	Provirus, SoloLTR	*gag, pol*	Barbulescu et al., 1999

10q24.2	ERVK-17, c10_B	**10**	(-)	101580569	101587716	Human Specific	N/A		*gag*	Macfarlane and Simmonds, 2004

11p15.4	K7	**11**	(-)	3468656	3478209	Human Specific^e^	15.44-27.95			Romano et al., 2006

11q12.1		**11**	(+)	58767448	58773196	Chimpanzee	N/A		*env*	This Study

11q12.3	K(OLDAC004127)	**11**	(-)	62135963	62150563	Gibbon^c^	19.46-35.24*		*pol*	Reus et al., 2001

11q22.1	K(C11c), K36, K118, ERVK-25	**11**	(+)	101565794	101575259	Human Specific	< 2	Provirus, SoloLTR	*pol*	Costas et al., 2001

11q23.3	K(C11b), K37, ERVK-20	**11**	(-)	118591724	118600883	Gorilla^b^	13.35-24.18		*gag*	Costas et al., 2001

12p11.1	K50e	**12**	(-)	34772555	34782217	Chimpanzee	39.23-71.02			Romano et al., 2006

12q13.2		**12**	(+)	55727215	55728183	Human Specific	< 2	Provirus, SoloLTR	*gag, pol*	Belshaw et al., 2005

12q14.1	K(C12), K41, K119, ERVK-21	**12**	(-)	58721242	58730698	Human Specific	< 2	SoloLTR	*gag, pol, env*	Costas et al., 2001

12q24.11		**12**	(+)	111007843	111009325	Human Specific	N/A			Medstrand and Mager, 1998

16p11.2		**16**	(+)	34231474	34234142	Unknown^d^	N/A			This Study

19p13.3	ERVK-22	**19**	(+)	385095	387637	Orangutan^c^	N/A			This Study

19p12a	K52	**19**	(+)	20387400	20397512	Orangutan^c^	29.71-53.79*			Hughes and Coffin, 2001

19p12b	K113	**19**	(-)	21841536	21841542	Human Specific	< 2	Provirus	*gag, pol, env*	Turner et al., 2001

19p12c	K51	**19**	(+)	22757824	22764561	Orangutan^c^	12.96-23.47*			Hughes and Coffin, 2001

19q11	K(C19), ERVK- 19	**19**	(-)	28128498	28137361	Human Specific	N/A	Internal Polymorphis m	*gag, env*	Tonjes et al., 1999

19q13.12a		**19**	(-)	36063207	36067434	Orangutan	N/A			This Study

19q13.12b	K(OLDAC012309), KOLD12309	**19**	(-)	37597549	37607066	Gibbon^c^	22.87-41.42*			Reus et al., 2001

19q13.42	LTR13	**19**	(+)	53862348	53868044	Orangutan	N/A		*env*	This Study

20q11.22	K(OLDAL136419), K59	**20**	(+)	32714750	32724384	Rhesus	15.74-28.5			Hughes and Coffin, 2001

21q21.1	K60, ERVK-23	**21**	(-)	19933916	19941962	Human Specific	3.46-6.27			Kurdyukov et al., 2001

22q11.21	K101, K(C22), ERVK-24	**22**	(+)	18926187	18935307	Human Specific	1.84-3.34			Barbulescu et al., 1999

U219	K105	**Unknown**	(+)	175210	176178	Gorilla^b^	8.74-15.82	Provirus, SoloLTR		Barbulescu et al., 1999

Yp11.2		**Y**	(-)	6826441	6833384	Chimpanzee	N/A			This Study

^a^Refers to most distant species with shared provirus. Determined by searching for provirus at the corresponding locus by BLAT searching flanking

sequence in the chimpanzee, orangutan, and rhesus publicly available genomes on the UCSC genome browser.

^b^From Macfarlane and Simmons, 2004.

^c^From Hughes and Coffin, 2001

^d^Provirus flanking sequence does not have a non-human primate orthologous location.

^e^Provirus is a product of a duplication event post-human/chimpanzee split.

^f^Ages based upon molecular clock calculations from Hughes and Coffin, 2005. N/A indicates unpaired LTRs or LTRs lacking sufficient sequence to determine age. Asterisk denotes age is listed for a recombinant provirus.

**Figure 1:**
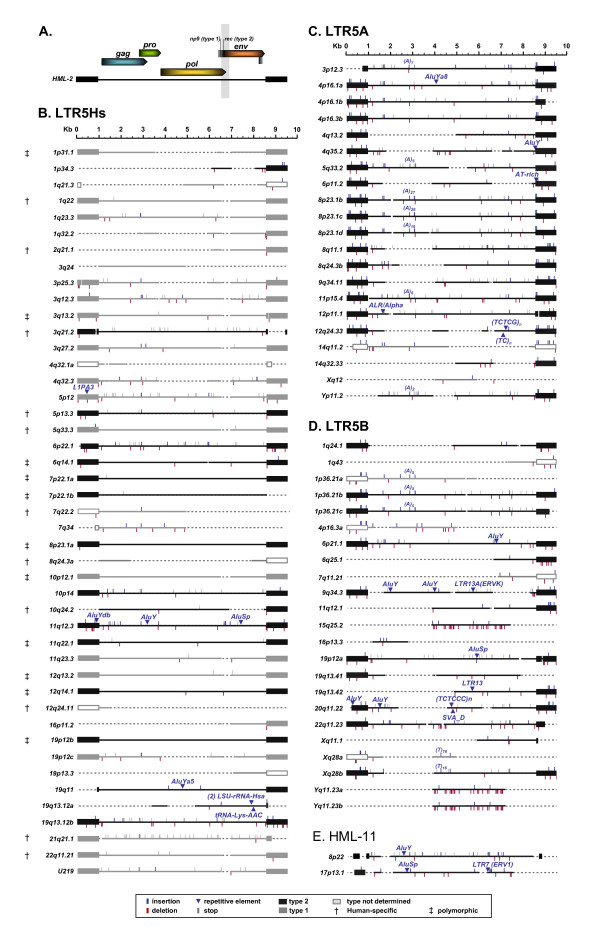
**Cartoon schematic of HML-2 proviruses in the human genome**. (A) A cartoon depicting the layout of the prototypical HML-2 retrovirus, including *gag*, *pro*, *pol*, and *env *gene positions. Splice sites of *env*, *np9*, and *rec *genes are also shown, with a faint gray band indicating the type 1 deletion region. Proviruses of the LTR5Hs (B), LTR5A (C), and LTR5B (D) groups are depicted and color-coded according to type. Type 1 proviruses colored in grey (with LTRs filled grey), type 2 colored in black (with LTRs filled black), and unclassified having open LTRs colored grey. Insertions and deletions < 3 bases are depicted with blue and red flags respectively. Larger insertions of retroelements are labeled according to type of element inserted, and large deletions are shown with dashed lines corresponding to missing sequence. Stop codons are indicated with a grey flag. Daggers indicate human-specific proviruses, with double daggers indicating polymorphic proviruses.

We expanded our original search to detect HML-2 proviruses that did not yield a high identity to full length K113, but still belonged to the HML-2 group. Using BLAT, we identified individual *gag*, *pro*, *pol*, and *env *genes and partial genes related to K113 in the GRCh37/hg19 build with the criterion for provirus identification as having at least one LTR associated with internal coding sequence in the same orientation. This search recapitulated our initial BLAT hits from the full-length K113 sequence and led to the cumulative identification of 17 additional elements (Table 2). Finally, we included an additional 8 elements not identified in BLAT searches but which have been classified according to RepeatMasker within the UCSC Genome Browser as "HERV-K", indicating HML-2 provirus sequence (bolded in Table 2), for a sum of 91 HML-2-related full-length and near full-length proviruses in the human genome. The inferred structure of each identified provirus is shown schematically in Figure 1, in which the length, insertions and deletions, ORFs, and introduced stop codons are indicated for each provirus relative to the K113 nucleotide sequence. The full-length nucleotide alignment is provided in Additional File 1. In all, we have generated an exhaustive dataset for the HML-2 group, adding 37 proviruses to the most recent report [52], around 30 of which are described here for the first time [5,8,11,15,51,53-55].

**Table 2:** Other HML-2 (and HML-2-like) proviruses

Provirus	Alias	Chr	Orientation	Start	End	Oldest Common Ancestora	Estimated Age^g^	ORFs	Reference
1p36.21a		**1**	(-)	12840260	12846364	Orangutan	N/A	*gag*	This Study

1p36.21c	K6,K76	**1**	(+)	13678850	13688242	Orangutan	22.69-41.09	*gag*	Hughes and Coffin, 2001

1p34.3		**1**	(-)	36955490	36956728	Orangutan	N/A		This Study

1q43		**1**	(-)	238925595	238927773	Rhesus	N/A		This Study

4p16.3b	K77	**4**	(-)	3980069	3988631	Chimpanzee	11.1-20.1		Romano et al., 2006

4p16.1b	K50c	**4**	(+)	9659588	9668650	Chimpanzee	17.19-31.13		Macfarlane and Simmonds, 2004

5q33.2	K18b	**5**	(-)	154016502	154024214	Chimpanzee	13.03-23.6	*env*	Romano et al., 2006

6p11.2	K23	**6**	(+)	57623896	57628704	Orangutan	9.38-16.99		Romano et al., 2006

7q11.21		**7**	(-)	65469689	65472384	Chimpanze^e^	N/A		This Study

**8p22**		**8**	(-)	**17765202**	**17773940**	**Rhesus**	N/A		This Study

8q24.3b	K29	**8**	(-)	146246648	146254211	Gibbon^f^	12.33-22.33	*env*	Hughes and Coffin, 2001

12q24.33	K42	**12**	(-)	133667120	133673132	Orangutan	7.07-12.81		Romano et al., 2006

14q11.2	K(OLDAL136419), K71	**14**	(-)	24480625	24484121	Orangutan	11.98-21.69	*gag*	Reus et al., 2001

14q32.33		**14**	(+)	106139659	106142540	Rhesus	N/A		Romano et al., 2006

**15q25.2**		**15**	(+)	**84829020**	**84832364**	**Orangutan**	N/A		**This Study**

**16p13.3**	**K(OLDAC004034**	**16**	(+)	**2976160**	**2977661**	Rhesus	N/A		**This Study**

**17p13.1**		**17**	(+)	**7960357**	**7967219**	Rhesus	N/A		**This Study**

19q13.41		**19**	(-)	53248274	53252591	**Orangutan**	N/A	*pol*	Hughes and Coffin, 2001

22q11.23	K(OLDAP000345), KOLD345	**22**	(+)	23879930	23888810	Gorilla^f^	21.64-39.18*	*gag*	Hughes and Coffin, 2001

**Xq11.1**		**X**	(+)	**61959549**	**61962054**	**Unknown^b^**	**N/A**	*env*	**This Study**

**Xq12**		**X**	(-)	**65684132**	**65686184**	**Human Specific^c^**	**N/A**		**This Study**

Xq28a	K63	**X**	(+)	153817163	153819562	Human Specificd	**N/A**	*gag*	Macfarlane and Simmonds, 2004

Xq28b	K63	**X**	(-)	153836675	153844015	Orangutan	14.65-26.52	*gag*	Macfarlane and Simmonds, 2004

**Yq11.23a**		**Y**	(-)	**26397837**	**26401035**	**Chimpanzee^e^**	**N/A**		**This Study**

**Yq11.23b**		**Y**	(+)	**27561402**	**27564601**	**Chimpanzee^e^**	**N/A**		**This Study**

^a^Refers to most distant species with shared provirus. Determined by searching for provirus at the corresponding locus by BLAT

searching flanking sequence in the chimpanzee, orangutan, and rhesus publicly available genomes on the UCSC genome browser.

^b^Region is flanked by satellite DNA, no good match found in non-human primate genomes.

^c^No good match found in non-human primate genomes.

^d^Provirus is a product of a duplication event post human/chimpanzee split.

^e^Provirus is a product of a duplication event post chimpanzee/orangutan split.

^f^From Hughes and Coffin, 2001.

^g^Ages based upon molecular clock calculations from Hughes and Coffin, 2005. N/A indicates unpaired LTRs or LTRs lacking sufficient sequence to determine age. Asterisk denotes age listed is for a recombinant provirus.

We conducted a separate analysis to identify HML-2 solo LTRs within the published genome. Solo LTRs are generated via recombination between the LTRs of a single provirus, or through recombination between different proviruses. Although solo LTRs have lost the ability to express viral gene products, their inherent promoter activity can affect expression of neighboring genes [9,56-59]. In general, estimates for the total number of HML-2 solo LTRs in the published genome have varied widely, from an original prediction of > 2500 [12] to the most recent figure of ~1200 [60]. We used the UCSC Genome Browser RepeatMasker algorithm, in which HML-2 elements have been assigned within the published sequence by nucleotide similarity to RepBase definitions of HML-2 LTRs. Using our approach, we identified 944 intact and nearly-intact HML-2 solo LTRs within the GRCh37/hg19 build (excluding 3 solo LTRs that represent polymorphic proviruses K105, K103, and 12q13.2) and used them to further characterize the HML-2 group of HERVs (Additional File 2).

### HML-2 subtype classification

HERV-K (HML-2) proviruses have been classified by the presence (type 1) or absence (type 2) of a 292 bp deletion at the *pol*-*env *junction [13]. Type 2 proviruses encode the accessory protein Rec, involved in the transport of unspliced mRNAs from the nucleus to the cytoplasm and analogous to the HIV Rev and HTLV Rex proteins [61-63]. The *rec *alternative splice site is deleted within type 1 proviruses (as is a portion of the *env *reading frame), resulting in mRNAs for a ~9 kDa fusion protein referred to as Np9 (Figure 1A) [30,64]. Within the HML-2 group, the frequency of type 1 proviruses was previously estimated to be around 44%, based on just 35 elements [11]. Of the 91 proviruses analyzed here, we could conclusively assign 75, of which 20 (~26%) were type 1 and 55 (~74%) were type 2 (Figure 1). The remaining 16 proviruses contained larger deletions spanning the Δ292bp *pol*-*env *feature used to differentiate the HML-2 subtypes and could not be classified. Two proviruses, Xq28a and 1p36.21a, are duplications of Xq28b and 1p36.21b/c respectively, but neither has retained the 3' ends due to truncating mutation. For these particular elements, we propose that the 1p36.21a and Xq28a proviruses are type 2 due to the fact that they are duplications of other type 2 proviruses.

We next analyzed type 1 and 2 frequencies with respect to HML-2 LTR subgroups (Figure 1B, C, and 1D). HML-2 LTRs cluster into one of three subgroups based on phylogeny and shared nucleotide features: LTR5Hs, LTR5A, and LTR5B [11,14]. In general, the LTR5Hs represent the most recent germline integrations and are the only subgroup with human-specific proviruses while the other subgroups are from older integrations. We observed that type 1 proviruses were exclusively found in the LTR5Hs subgroup and not in the LTR5A or LTR5B subgroups (Figure 1). Of the 45 LTR5Hs proviruses, types 1 and 2 were roughly equally represented, with 20 (~44%) and 17 (~38%) elements, respectively (Figure 1B). By contrast, all of the analyzable LTR5B (17 proviruses, or ~84% of the subgroup) and LTR5A (19, or ~90%) proviruses were type 2 (Figures 1C and 1D).

### LTR-based analysis of HML-2 proviruses

Phylogenetic analysis of the LTRs of endogenous proviruses not only reveals their individual relationships and grouping, but also provides useful insight into their evolutionary history. Since the LTRs of a provirus must be identical in sequence at the time of integration, terminal branches separating the 2 LTRs of each provirus on such a tree reflect accumulated mutations since the time of integration, whereas internal branches reflect evolution (mostly as a virus) prior to the final germline integration. Violations of this pattern reflect genomic rearrangements, such as gene conversion and recombination [51,65,66]. To investigate the overall branching patterns and evolutionary dynamics within the HML-2 group, we performed a phylogenetic analysis using Bayesian inference of the LTRs associated with individual proviruses (data not shown). In all, this analysis included LTRs from all proviruses identified, excluding the seven without an associated LTR. The resulting phylogeny revealed two major lineages, the first containing the most recently formed proviruses with the longest internal branches and shortest terminal branches, including all human-specific members and those with known polymorphic alleles. The second lineage included evolutionarily older proviruses, many of which are shared among most primates [14,51]. Our tree topology is consistent with previous reports [11,14,18,51] but includes at least twice the number of provirus-associated LTRs, and thus provides a more detailed representation for evolutionary analyses of the HML-2 HERVs.

**Figure 2:**
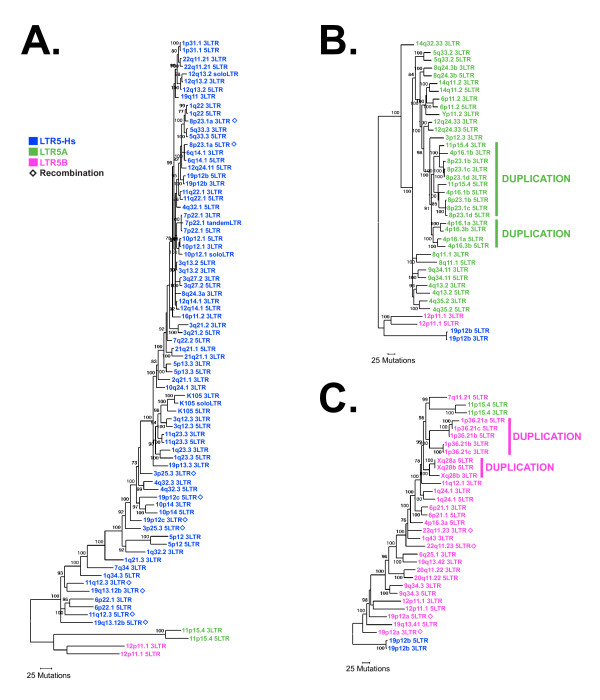
**Phylogeny of provirus LTR sequences**. Bayesian inference trees were generated using 5' and 3' LTRs of HML-2 provirus elements in the human genome. LTR sequences of less than 250 bases in length were not included, as they limited capacity to detect phylogenetic relationships among LTR sequences. Sequences are color-coded according to distinctive LTR subgroup features (see Methods). LTR5Hs sequences are shown in (A), with 5B and 5A sequences added to serve as a reference. LTR5A (B) and LTR5B (C) are similarly displayed. Open diamonds indicate recombinant proviruses, and duplications are grouped using colored bars. Posterior probability values > 70 are shown for the best tree rooted on 5A and 5B (A), 5Hs (B), and 5B and 5Hs (C).

Overall, our classification largely agrees with the previous report defining the three major subgroups [14]; however, the larger sample size of our data set highlighted inconsistencies in the previous classification system. For example, the 8q11.1 and 4q13.2 proviruses are each characterized by RepeatMasker as full-length elements with LTRs belonging to different subgroups: 8q11.1 having a 5' LTR5B and 3' LTR5A, while the opposite was reported for 4q13.2 (5' LTR5A and 3' LTR5B). We found that the direct repeats flanking the edges of each provirus were intact and identical, suggesting that the proviruses were not generated through recombination. Moreover, the LTRs from each provirus grouped as nearest neighbors (Figure 2C), and inspection of each LTR revealed sequence features consistent with LTR5A. Thus, there is a need to reclassify the LTR subgroups based upon our phylogeny.

To reclassify LTR subgroups, we added sequence from 944 HML-2 solo LTRs to our initial proviral LTR alignment. Individual elements were initially categorized as predicted by RepBase [67] and subsequently re-analyzed by sequence comparison to the group consensus. In a sequence comparison of 1092 HML-2 LTRs, we successfully identified subgroup-specific features which we used to discriminate the LTR5Hs, LTR5A, and LTR5B elements. We would like to note that previously described sequence polymorphisms [14] were not observed among all sequences in any subgroup, likely due to our large sample size. With reference to the full-length alignment (Additional File 1), we identified a 4 bp insertion at positions 585 and 10718 in > 80% of all LTR5Hs, an insertion at positions 806 and 10957 shared in > 99% of LTR5A and B, and insertions unique for all LTR5A elements seen at positions 182 and 10317.

Using these subgroup-specific features, we classified individual proviruses and analyzed their distribution within the HML-2 group (Figure 2). The largest lineage represented the more recent LTR5Hs subgroup (Figure 2A) with 45 proviruses (~50%). Of the other subgroups, 21 LTR5A (~24%) and 23 LTR5B (~26%) proviruses were found (Figures 2B and 2C, respectively). We hypothesized that each subgroup would have arisen independently, so we attempted to formulate phylogenetic trees of each subgroup using the other subgroups as outgroups. This approach was successful for LTR5Hs and LTR5A trees (Figures 2A and 2B), but not for LTR5B (Figure 2C). As seen in Figure 2C, a tree of LTR5B sequences rooted on LTR5Hs sequences has 5A sequences nested within the 5B sequences (and vice versa for a tree rooted on 5Hs sequences). We concluded that the LTR5B subgroup is the oldest (Figure 2B) and ancestral to the other two subgroups, each of which arose independently and uniquely from viruses of the LTR5B group.

**Figure 3:**
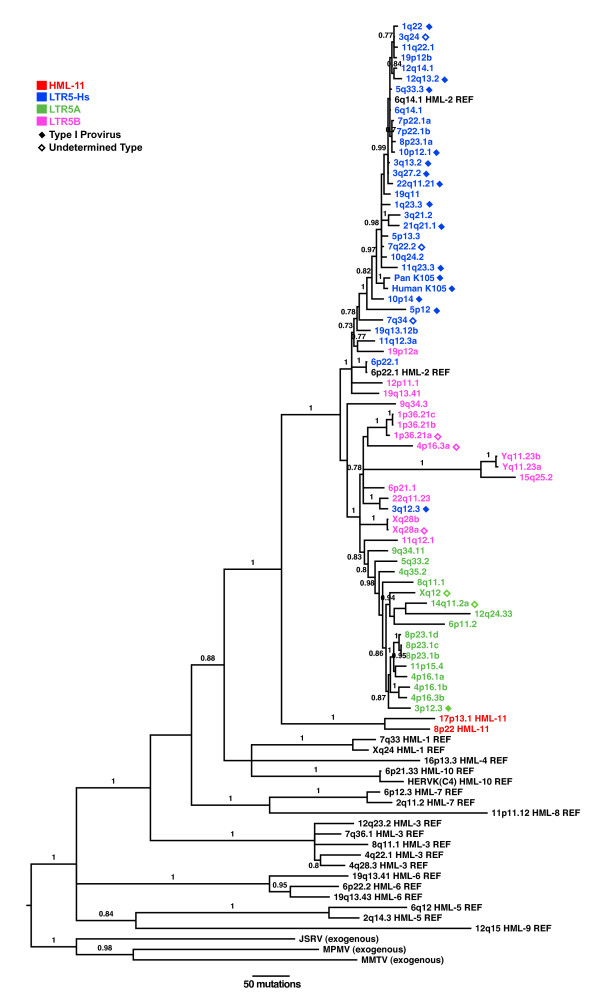
**Phylogeny of HML endogenous elements**. A Bayesian inference tree of the *pol *gene from prototypical members of HML 1-10 families (indicated by "REF") along with exogenous betaretroviruses MMTV, MPMV, and JSRV was generated to characterize proviruses identified through our BLAT search (black). Sequences were colored according to LTR5 subgroup and annotated with filled diamonds for type 1 proviruses and open diamonds for proviruses of undetermined type. Colored sequences without diamonds represent type 2 proviruses. Posterior probability values > 70 are shown for the tree rooted on the exogenous betaretrovirus sequences.

**Figure 4:**
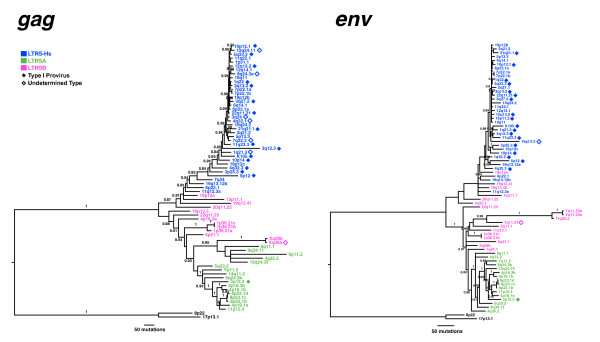
**Phylogenetic analysis of *gag *and *env *genes**. Bayesian inference trees were generated for the first ~1800 bp of *gag *(A), as well as the SU portion of *env *(B). Trees were rooted using the 17p13.1 provirus sequence as an out-group, with posterior probabilities above 80 shown. Sequences are color-coded according to LTR group, with type 1 proviruses indicated with filled diamonds, and undetermined types with open diamonds. All other proviruses shown are type 2.

The LTR5A subgroup of proviruses contains a well-supported clade comprising two clusters, the first represented by proviruses at 4p16.1b, 8p23.1b, c, and d, and 11p15.4, and the other by 4p16.1a and 4p16.3b (labeled in Figure 2B). The 5' LTRs within each cluster group together, as do the 3' LTRs, suggesting an initial duplication of a single integrated provirus to sites on different chromosomes, each of which was subsequently amplified locally to generate each cluster. Overall, the elements within this clade share several sequence features, such as short insertions/deletions and single base changes relative to K113 (indicated in Figure 1 and 1detailed in Additional File 1), and have high overall nucleotide identity (96.2% within the group). More specifically, the 8p23.1b, c, and d proviruses exceed 99.9% identity, and are more than 95% identical to the 11p15.4, 4p16.1, and 4p16.3 proviruses. Not surprisingly, we observed > 1kb of cellular DNA flanking each provirus shared the same level of nucleotide identity (data not shown), demonstrating that these elements arose, at least in part, via repeated large-scale duplication, most likely mediated by some element outside the provirus itself. Clustering within the *pol*-, *gag*- and *env*-based trees also lends support for the subgroup's history (Figures 3 and 4). Also within the LTR5B group, a similar pattern is observed, specifically leading to the Xq28a and b, and the 1p36.21a, b, and c clusters (also indicated in Figure 2C).

### Gene-based phylogenies of HML-2 proviruses

We analyzed all identified proviruses phylogenetically to support their classification in the HML-2 group, and to determine their relationship to one another and more distantly related HERV-K elements. In the tree shown in Figure 3, we compared a region of the *pol *gene present in most of the proviruses in our dataset (65 in all) with representative members of HERV-K groups HML-1 through HML-10 and the exogenous betaretroviruses: mouse mammary tumor virus (MMTV), Mason-Pfizer monkey virus (MPMV), and Jaagsiekte sheep retrovirus (JSRV). Within the *pol*-based phylogeny, the HML-2 proviruses form a distinct lineage (Figure 3), with the most recent integrations near the tips of the tree and consistent with the observed phylogeny based on the LTRs (Figure 2). Superimposition of the HML-2 subtypes onto each phylogeny revealed a skewed distribution of type 1 proviruses, which are predominantly within the LTR5Hs group (filled diamonds), also consistent with our initial subtype classification (Figure 1). The observed polyphyletic distribution of type 1 proviruses as previously reported [11,14], might reflect gene conversion events post-integration [13], or could be due to rescue of the inherently noninfectious type 1 genomes by coexpressed type 2 proviruses followed by frequent recombination between the two. However, the lack of gene conversion in 5Hs LTRs and the frequency of type 1 integrations in the last 5 million years suggest that exogenous recombination prior to integration in the germ line is the most likely explanation.

To account for the remaining HML-2 sequences not included in the *pol*-based phylogeny, we performed a Bayesian inference analysis using the first ~1.8 kb of the *gag *reading frame as well as the first ~1 kb of *env *corresponding to the SU region (Figure 4). In each phylogeny, the proviruses we identified grouped together into a well-supported clade with other previously described HML-2 elements. There were two exceptions, namely for proviruses located at 8p22 and 17p13.1, which together formed a distinct group within the *pol*-based tree. Both were classified by RepeatMasker as HML-2, and have been included in our current HML-2 dataset (Table 2); however, we speculate that the proviruses represent a previously unrecognized HML group. It is unlikely that these elements arose out of recombination of HML-2 elements with non-HML-2 elements, as the dissimilarity exists throughout the genome of these proviruses. With the exception of 17p13.1 and 8p22, we were unable to align non-HML-2 betaretrovirus-like sequences to either the SU or *gag *regions of HML-2, providing further support that 89 proviruses identified belong exclusively to HML-2. Though 17p13.1 and 8p22 were capable of being aligned, they were found to be so distant in sequence that they cannot represent HML-2 elements, but instead must be representative of a group of proviruses that is a close cousin to HML-2, which we term HML-11.

Interestingly, the LTR5Hs provirus located on 3q12.3 was found to be most similar on the *pol *tree to 22q11.23, an LTR5B provirus (Figure 3). However, when compared to *gag *and *env *sequences (Figure 4), 3q12.3 grouped with the LTR5Hs sequences, and it would appear that it is a recombinant provirus within the *gag *and *pol *genes. We identified break points between positions 2710 to 5102 with LTR5B proviruses as well as 5465 to 6390 (data not shown). A possible explanation of these results is that the 3q12.3 is an integration of a recombinant virus intermediate to LTR5Hs and LTR5B.

### Evolutionary dynamics of the HML-2 group

We, and others, have previously established that past recombination events between proviruses can be inferred through phylogenetic analysis of paired LTRs [18,51,65]. The LTRs of an individual provirus are identical at the time of integration and subsequently evolve independently. As a result, the 5' and 3' LTRs from a single provirus will be more similar to one another than those of any other element, and each pair will form a distinct phylogenetic group. Recombination after integration can be inferred by violation of this property, resulting in non-paired 5' and 3' LTRs for a given provirus. We examined the LTR-based phylogeny for evidence of recombination, with close attention to those proviruses that were previously unreported. We observed 6 examples of non-paired 5' and 3' LTRs, specifically from proviruses located at 3p25.3, 8p23.1a, 11q12.3, 19p12a, 19p12c and 19q13.12b (indicated by open diamonds at the branch termini in Figure 2), although not in the case of any of the previously uncharacterized HML-2 elements. The 6 proviruses with non-paired LTRs that we observed were similar in chromosomal location to those originally reported in 2001 [8,51], and we confirmed their identity by BLAT searching the nucleotide sequence of each provirus from our dataset to the earliest available human genome build (July 2003, NCBI34/hg16) (data not shown).

### Estimation of the relative ages of individual HML-2

Because the LTRs are identical at the time of integration, the number of differences between the LTRs can be used to infer the relative age of a provirus. The ages of HML-2 proviruses as a function of LTR subgroup were previously estimated by Buzdin *et al*. based on the intrabranch divergence between individual elements from the subgroup consensus. Their analysis of ~40 LTRs estimated that the LTR5A and 5B subgroups formed around 5.8 and 10.3 million years ago (mya), respectively [14], with 5A originating from 5B. These are fairly recent estimates for these subgroups, given that most LTR5A and 5B proviruses have shared loci among primates whose divergence from humans substantially predates this timeframe. This underestimation is likely due to faulty molecular clock assumptions as well as the use of relatively few proviruses from early sequence builds. Using the HML-2 elements within our dataset, we estimated the time of integration for each 2-LTR HML-2 element using a previously described method [66,68] in which divergence between LTRs is normalized to a standard mutation rate of 0.24-0.45% per million years. In total, we were able to place age estimates to 49 HML-2 proviruses, provided in Tables 1 and 2. We decided that we could not accurately determine the time of recent provirus integrations (those elements with lower bound estimates below 2 million years ago), so these proviruses are listed as "< 2" in Table 1. But it is worth mentioning that our age estimates are similar to those seen by Jha et al. [50,69]. On average, LTR5Hs proviruses were estimated to have formed ~9.1 mya for all proviruses: ~2.7 (± 1.1) mya for those specific to humans. Consistent with the LTR-based and internal-based phylogenies (Figures 2, 3, and 4) we found the LTR5A and LTR5B proviruses to have formed earlier, around ~20.1 (± 5.4) mya and ~27.9 (± 12.0) mya, respectively.

**Figure 5:**
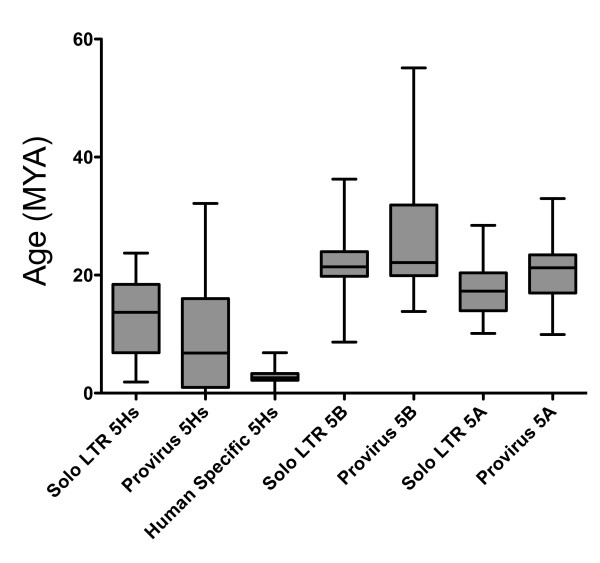
**Age of HML-2 LTRs**. LTRs from 5Hs, 5A, and 5B subgroups were used to determine distance measurements as a function of time of integration. Ages of "Solo LTR" elements were determined as described in Methods, while "Provirus" element ages were determined by 5' to 3' distance measurement. "Human Specific 5Hs" refers to solo LTRs that are only found in the human genome, and whose age was calculated using the solo LTR age calculation method.

We developed a new algorithm to calculate age of solo LTRs and proviruses with only one LTR. We grouped LTRs based on the subgroup-specific features described above, and then determined the divergence of each LTR to a subgroup consensus. This was then normalized using an average mutation rate (0.34% per million years) as for the provirus molecular clock. Ages for each solo LTR element can be seen in Additional File 2. We were initially concerned that our solo LTR age calculation would be biased by mutations between proviruses prior to integration or as a function of recombination to produce the solo LTR elements. However, when we compared the age calculation of solo LTR elements for each subgroup ("Solo LTR", Figure 5) to provirus age calculations ("Provirus", Figure 5), we found comparable age distributions. Furthermore, when we performed comparative genomics on a subset of solo LTR elements to determine whether our age calculation corresponded to presence of the element in the appropriate genomes we found an accuracy of 50-60% using our solo LTR age calculation algorithm. In contrast, when we examined distribution of proviruses whose age was determined using the 5' to 3' divergence method in the appropriate genomes, we only had an accuracy of 40%. Together this supports our approach to calculate age of solo LTR elements in the genome, and implies that there is little divergence within a subgroup of closely related proviruses at time of integration. This methodology will enhance our ability to study the chronology of endogenous retrovirus integration in the genome.

Comparing the relative ages of LTRs revealed several trends in the evolutionary activity within each subgroup (Figure 5). We report for the first time that LTR5Hs represents a broad subgroup that has continuously been integrating into the germline for the last 20 million years. We found that 5Hs provirus integrations predominantly (~50% of all integrations) occurred between 6-18 mya. The trends we observed for the LTR5Hs elements were in contrast to the patterns of activity we saw for the LTR5A and LTR5B subgroups, which have a narrower timeframe of activity, with the LTR5A ranging from ~15-21 mya, and LTR5B from ~19-25 mya. This observation is consistent with LTR5Hs retaining activity throughout primate evolution, and the LTR5A and LTR5B subgroups having become extinct prior to the *Homo *divergence. Overall, these data provide an estimate for individual HML-2 provirus formation, and suggest that the subgroups co-existed at least during the early evolution of primates, but that only the LTR5Hs group retained access to the germline along the lineage leading to humans. Although it has been proposed that some HML-2 elements are still active as viruses [29], none have been found to date.

### Maintenance of HML-2 elements in the genome

We initially sought to determine the association of HML-2 elements with respect to gene regions, as we hypothesized that HML-2 viruses preferentially integrate in or near gene regions like MLV and HIV [70]. We found that ~60% of all elements were found in or within 30 kb of gene regions (data not shown). Interestingly, ~20% of all elements were present within introns inside genes and ~80% of those elements inside genes were in the antisense orientation, which corroborates the notion that HML-2 integrations inside genes are selected against unless they are present in an antisense orientation [60].

**Figure 6:**
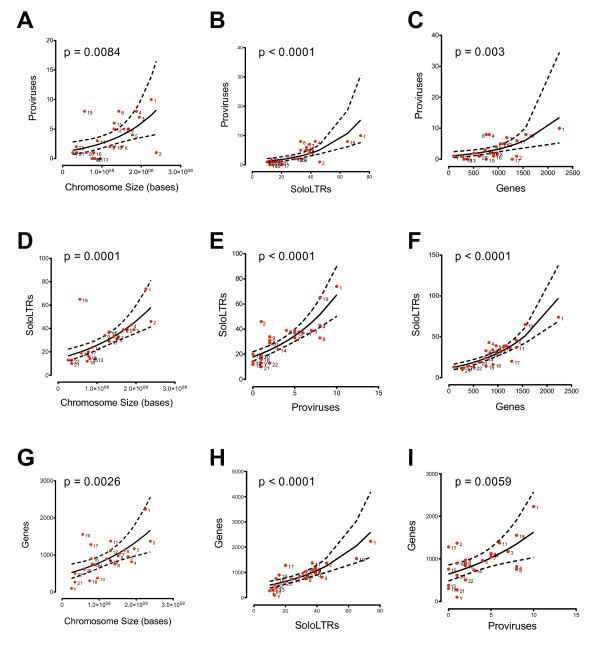
**Relationships between chromosome size, solo LTR, provirus or RefSeq gene frequency**. Binomial regression analysis was performed using chromosome size (A), solo LTR frequency (B), or RefSeq gene frequency (C) as a predictor of provirus frequency; or predicting solo LTR frequency using chromosome size (D), provirus frequency (E), or RefSeq gene frequency (F); or predicting RefSeq gene frequency using chromosome size (G), solo LTR frequency (H), or provirus frequency (I). The bold line represents mean correlation, with 95% confidence intervals shown with dashed lines. P-values are shown for each plot.

We wanted to determine whether the gene bias for HML-2 integrations in provirus or solo LTR form correlated with gene density on any given chromosome. To address this question, we investigated the respective distributions of HML-2 proviruses and solo LTRs as a function of chromosome size (total bases) or RefSeq gene density for each chromosome (Figure 6). Values for total proviruses and solo LTRs were from the HML-2 dataset presented in this study, and values for chromosomal size and gene density were extracted using the Base Position and RefSeq Genes tracks within UCSC Genome Browser, respectively. For each pairwise set, expected frequencies were predicted using a negative binomial regression analysis and compared to the observed frequencies using a χ^2 ^analysis (p-values provided in each panel in Figure 6). In general, our results indicate that, though statistically significant, chromosome size is a relatively weak predictor of the frequencies of proviruses, or gene density (Figures 6A and 6G). Also relatively weak is the relationship between gene density and provirus frequency per chromosome (Figures 6C and 6I). However, we did find provirus and solo LTR frequencies per chromosome (Figures 6B and 6E) to be relatively strong predictors of one another, as expected. We also observed a strong relationship between chromosomal gene density and solo LTR frequency (Figures 6F and 6H), but not for proviruses. A possible explanation for this discrepancy would be selection against endogenized proviruses with respect to genes forcing either conversion to solo LTR elements or loss from the genome.

Because our relationships describe each other well, we were able to observe that a few chromosomes were over- or under-represented in proviruses, solo LTRs, or RefSeq genes. This relationship was determined by identifying outliers of varying position and magnitude with respect to the regression. For example, our results indicate that chromosome 19 is particularly dense in both proviruses and solo LTRs (Figures 6A and 6D) as well as genes (Figure 6G). This correlation is consistent with a previously described trend of HERV over-representation on this chromosome, as well as preferential integration by HIV and MLV [70,71], a phenomenon suggested to result from multiple segmental duplications [72]. Interestingly, the same study found chromosome Y enriched in overall HERV content, however we did not observe this trend when considering HML-2 alone (Figures 6A and 6D). Other outliers include chromosome 4, which appears somewhat enriched in solo LTRs with respect to gene density, whereas chromosomes 15, 16, and 17 are deficient in solo LTRs in the same respect (Figures 6F and 6H). Interestingly, chromosome 17 is relatively gene-rich (Figure 6G), but is devoid of HML-2 proviruses and solo LTRs, implying a selective pressure against HML-2 integration or maintenance on this chromosome. This observation is in contrast to the trend we observed for chromosome 19 as discussed above (Figure 6G). As shown in Figures 6B and 6E, the equilibrium between proviruses and solo LTRs is a relatively strong fit (*p *< 0.0001 in either direction). Exceptions are chromosome 8, which is relatively provirus-rich compared to solo LTRs (Figure 6B), and chromosome 2, for which the opposite trend is observed (Figure 6E). Possibly, these outliers are indicators of specific chromosomes that either preferentially maintain proviruses (i.e., chromosome 8), or for which there exists a heavy selective pressure for recombination leading to solo LTR formation (i.e., chromosome 2). Though, it remains to be seen if these results are a general trend for HERV representation in the genome or if these are HML-2 specific results.

## Discussion

This report comprises the most complete and up-to-date analysis of HML-2 proviruses and solo LTRs that can be found in the published human genome. The HML-2 group comprises the most recent integrations of endogenous retroviruses in humans, and includes many members that are polymorphic within the species. It has been hypothesized that these endogenous elements represent the closest relatives of extant exogenous betaretroviruses that may retain the capacity to infect humans [16,18]. These putative exogenous viruses have a proposed role in breast cancer as well as biliary cirrhosis, although no such virus has been convincingly detected [73-75]. Based on expression patterns, many groups have also suggested a role for endogenous HML-2 proviruses in various diseases from breast, ovarian, and skin cancers, to schizophrenia and arthritis [21,24,28,34,39,44,76]; however, a functional link to these diseases also remains to be established. Despite the mounting evidence suggesting a clinically significant role for HML-2 proviruses in disease, it is surprising that no study has yet fully described all HML-2 proviruses in the published human genome. As it is unlikely that each provirus equally contributes to every disease to which HML-2 expression is associated, identification of which provirus is expressed in these diseases remains impossible unless all known proviruses are characterized.

Here, we have identified and characterized 91 provirus elements present in the human genome, adding almost 30 more than have been previously described. We have also identified 944 solo LTR elements, over 1500 fewer than previously expected in the human genome [12], and 300 fewer than the closest suggested estimate [60]. Discrepancies between our estimate of solo LTR number and that published previously [60] are likely due to our exclusion of elements that are not full length or near full length (> 750 bp). It is unlikely that this variance is due to differences in genomic builds, as when we compared the build used previously to the current build using the same criteria, we found the same number of solo LTR elements in both builds, and not the number reported previously (data not shown). While we believe our list is as comprehensive and as thorough as possible, it has excluded for the benefit of accuracy many partial provirus elements that lack sufficient sequence to determine their grouping within HERV-K elements. Also, there are at least 4 provirus elements not present in the human genome as full-length elements, due to their polymorphism. Therefore, it is very likely that 89 is an underestimation of the actual number of HML-2 proviruses, but it is the best approximation available to date. Previous groups have attempted to compile a list of HML-2 proviruses in humans, but the closest identification of all HML-2 proviruses in humans was only able to identify 54 proviruses, and many of the loci have changed since the publication of this report [52].

Because of the inherent ambiguity of genomic builds, it becomes increasingly important to develop a standard for HML-2 provirus identification. Previous publications reporting HML-2 proviruses in the human genome include accession numbers referencing these proviruses corresponding to BACs, which provide little to no useful information about the proviruses described. Therefore, comparing information on HML-2 proviruses becomes difficult as different groups use different nomenclature to define proviruses, and many use different accession numbers for the same provirus, as multiple BACs may contain the same locus. Finally, some accession numbers are for BACs that include more than one provirus [11,51]. This confusion can be reduced through a few measures that this study provides: 1) deposition of all HML-2 sequences identified and their flanking sequences into GenBank; 2) standardization of HML-2 nomenclature; 3) subclassification of HML-2 for functional studies; 4) thorough analyses of all HML groups to define criteria for what qualifies a new element to belong to an existing group. Here, we have created a database of HML-2 provirus and flanking sequence that has been deposited into GenBank as well as clearly defined properties for all known HML-2 elements.

Although attempts have been made to standardize nomenclature for HERVs using tRNA primer as defining characteristic [77,78], we believe that for the HERV-K elements this does not make sense. HML-5 has a sequence that suggests priming from a Met tRNA, suggesting it belongs to a HERV-M group, despite being closely related to HERV-K [77]. Many elements may lack or have mutated primer-binding sites precluding it from classification using this system. Therefore, while we do believe it is necessary for having a standardization of nomenclature for HERVs in the genome, we propose that all betaretrovirus-like elements be identified as HML-X (where "X" is 1-11, based upon phylogenetic similarity to known HML groups) followed by their locus on the human chromosome. An example from this study would be HML-2(3q12.3). While this nomenclature is limited to human genomes, it does provide a useful reference point when analyzing betaretrovirus-like ERVs in non-human primates. Further work is necessary for defining properties of all endogenous retroviruses in the human genome.

It seems likely that most HML-2 proviruses are the result of independent integration events that have been preserved within the genome. However, there are 17 elements that are in the genome as a consequence of transposition events that include both a complete provirus and at least 1500 kb of flanking DNA. Based upon our estimates, these proviruses have been in the genomes of primates for 20-30 million years, though it is likely that these transpositional events occurred very recently, approximately around the split of humans and chimpanzees (~5.5 mya [68]). This is seen by the incomplete expansion of elements 8p23.1d and 11p15.4 which do not have a corresponding provirus in chimps, while 8p23.1c and 8p23.1b do. The expansion of elements in the Xq28 locus corresponds to gene duplication of the cancer testis antigen 1 (CTAG1) into CTAG1A and CTAG1B; both CTAG1A and CTAG1B are exclusively expressed in malignant tissues or normal testis [79], which is the same expression pattern of HML-2 proviruses. This gene duplication is present in the chimpanzee, human, and orangutan published genomes, but not rhesus genome, consistent with the estimated integration time of the Xq28b provirus (~21 mya). The duplications in 1p36.21 are found within the PRAMEF gene cluster, comprising genes that are closely related to PRAME, another gene that is exclusively expressed in malignant tissues or normal testis [80]. The duplicated 5A elements are all flanked by hypothetical proteins, therefore it remains to be seen what the significance of this expansion is. Nevertheless, it is interesting that the same element, along with flanking sequence was transposed multiple times, while most other elements were not; this strongly implies that the transposition is due to some element in the flanking DNA, not the provirus itself. This pattern contrasts with the ERV9 family of endogenous retroviruses, which have continued retrotranspositional activity within the genome since the hominid divergence within the primate lineage [81].

We have reclassified the different subgroups of HML-2 proviruses based upon unique signatures of our 1087 HML-2 LTR sequences (947 solo LTRs and 140 provirus-associated LTRs). We did not observe the sequence polymorphisms within subgroups of our sequences as previously used to define the groups [14], likely due to our much larger sample set. However, we did observe an LTR5Hs-specific 4 base insertion at position 585 and 10718 of the HML-2 provirus alignment (Additional File 1), which was found in ~80% of all LTR5Hs proviruses. LTR5A/Bs also have a unique insertion at positions 806/10957, which is found in all LTR5A/B sequences, but none of LTR5Hs. Furthermore, LTR5A can be identified by unique insertions at positions 182/10317. All of the figures in this publication are reflections of our definitions of LTR grouping, rather than previously inaccurate groupings. It should be noted that our reclassification of LTR is significant in categorizing viruses, as all of our phylogenetic trees (Figures 3 and 4) of provirus genes confirms monophyly of subgroups. As such, we feel our method of grouping LTRs is a rigorous and predictive method to identify HML-2 elements in future sequenced genomes.

It is of interest that proviruses and solo LTRs appear to have been differentially maintained within the genome. Under a neutral model of evolution, one would imagine that there should be approximately the same proportion of proviruses and solo LTR elements to size of chromosome or gene density of any given chromosome. In general, this principle holds true, though four chromosomes stand out - chromosomes 2, 4, 8, and 17. While chromosomes 2 and 17 are gene rich, they are relatively devoid of both proviral and solo LTR elements. Conversely, chromosomes 4 and 8 are seemingly enriched in HML-2 elements compared to RefSeq genes. Furthermore, we observed an enrichment of proviruses compared to solo LTRs on chromosome 8, and an enrichment of solo LTRs compared to proviruses on chromosome 2. A possible explanation for this would be that human chromosome 2 is a product of fusion of two smaller chromosomes in other primates. When this fusion event took place, it is conceivable that the recombination of many highly similar DNA sequences occurred leading to production of more solo LTRs than proviruses on this chromosome. It is difficult to determine if this is the case as most non-human primate genomes are unfinished and many proviral loci are not assigned to any given chromosome. Initial analysis identified one provirus on Chromosome 2a and 2b in chimpanzee and at least 5 proviruses in orangutan (data not shown). Further drafts of non-human primate genomes are necessary for this type of analysis to be performed in other species.

The significance of the increased ratio of proviruses to solo LTRs on chromosome 8 is unclear, although the distribution may simply be skewed by the expansion in the 8p23.1 locus. Removal of two proviruses on chromosome 8 puts the point within the 95% confidence interval of the solo LTR-provirus correlation. Nevertheless, our study shows that endogenous retroviruses can be used to study genome evolution, as they are present in numbers sufficiently large to be informative, but much more manageable than SINE or LINE elements. Finally, the strong correlation of HML-2 elements and gene regions, may reflect a propensity to integrate in such regions [71], or, conceivably some sort of protection against mechanisms designed to remove transposable elements [82]. However, this conclusion may be an oversimplification of a more complicated mechanism of regulating repetitive elements within the genome. The fact that so many elements are maintained in or near genes may provide a partial explanation for the correlation of HML-2 gene expression with various disease states.

While disease association of HML-2 proviruses is controversial, many believe that HML-2 expression in diseased tissue is a byproduct of cellular dysfunction. Others have argued that exogenous retroviruses may recombine with homologous endogenously expressed HML-2 elements yielding infectious viruses that cause disease. This study is also the first to thoroughly identify and characterize all available human HML-2 proviruses. Correlation of HML-2 expression to disease onset is well-supported, and suggests that provirus expression may be a useful biomarker for certain diseases, particularly breast cancer, where no useful diagnostic marker currently exists [83]. Here, we have provided a list of provirus open reading frames (Tables 1 and 2) that may represent putative targets for detection of disease using HML-2 proteins or mRNA transcripts as biomarkers. We are also making available complete files of the sequences identified through deposition in Genbank (accession numbers: JN675007-JN675097) along with flanking sequences (accession numbers: JN675098-JN675187). Finally, we have aligned these sequences (Additional File 1) and proved them as a useful reference that can be viewed using any sequence viewing software. These steps should prove helpful in identifying and categorizing HML-2 expression in disease and assigning sequences detected to specific proviruses and, therefore, chromosomal locations.

Two genes not analyzed for expression are *np9 *and *rec*, alternative splice products of type 1 and type 2 env genes, respectively. Although *rec *transcripts are found in normal and cancer tissues, *np9 *mRNA has only been detected in tumor tissue, as is observed in tissues from mammary carcinoma biopsies, suggesting a possible role in tumorigenesis [64,84,85]. The type 1 proviruses all belong to the LTR5Hs subgroup, the most recent subgroup of HML-2 elements in the genome. Six of the 20 type 1 proviruses contain open reading frames for the *env *gene without having the 292 bp sequence for expressing functional Env. It is possible that the retention of an open reading frame in the remaining *env *sequence plays some role in the disease association of HML-2 proviruses.

The observations that type 1 proviruses are found almost exclusively within the LTR5Hs group of proviruses but are not monophyletic, combined with their patent incompetence for independent replication, are most consistent with their arising repeatedly by gene conversion of existing proviruses or by recombination between genomes arising from replication competent type 2 proviruses during reverse transcription prior to integration. Of these two models, recombination during reverse transcription is by far the more likely. First, if gene conversion post-integration was so frequent, it would also be seen in other parts of the genome, particularly in the LTR, where it is readily detected [53,65,66]. However, such events, although they can be detected over evolutionary time, are quite infrequent for HML-2 proviruses [86]. By contrast, recombination during reverse transcription of copackaged RNA genomes is the rule during retrovirus replication, and averages of 5-10 crossovers per genome per replication cycle have been estimated. Since all initial integrations almost certainly arose from infection of the germ line by an HML-2 virus produced by a somatic cell, which also contained and expressed type 1 proviruses, the heterozygous virions necessary for recombinant formation would have been very frequent, and such recombinants would arise at high frequency. An interesting topic for speculation is whether the deletion itself or the Np9 protein that results from it promotes this process in some way, for example by causing higher levels of expression of type 1 genome RNA.

The polymorphic nature of HML-2 proviruses may play an important role in the polymorphism of diseases with which they are associated. Genome-wide association studies (GWAS) have proven very useful for correlating single nucleotide polymorphisms (SNPs) to various diseases [87]. We attempted to determine if there were any proviruses or solo LTR elements present between SNPs shown to be involved in disease; however, we did not identify any proviruses that were linked to disease-associated SNPs (data not shown). This result does not preclude the possibility of association of polymorphic proviruses not present in the published genome with these SNPs. Also, many SNPs found on repetitive elements like proviruses are precluded from GWAS analysis, thereby eliminating the possibility of studying disease association of polymorphic proviruses. The abundance of solo LTRs and proviruses in close proximity to genes would indicate that there is some protection for these elements within the genome. For that matter, dysregulation of solo LTR formation and recombination of proviruses might play an important role in disease.

## Conclusions

Our analysis of the completed published human genome sequence has identified 89 HML-2 elements, over 30 more than previously described, as well as a new group of HML endogenous retroviruses (HML-11). We have catalogued and estimated the time of integration of these elements as well as providing an algorithm to identify time of integration of almost 1000 solo LTR elements. Integration of HML-2 elements appears to have occurred and been preserved in or near gene regions, much like MLV and HIV. Our analysis has restructured the classification of HML-2 elements and provides a useful tool for the future analysis of human endogenous retroviruses in evolution as well as their role in human disease.

## Materials and methods

### *In silico *identification of HML-2 proviruses and solo LTR elements

To identify the chromosomal coordinates of HML-2 proviruses in human DNA, we searched the most recent genome build (GRCh37/hg19, February 2009) using the UCSC BLAT program [49] for sequences related to the full-length nucleotide sequence of the K113 provirus (AY037928) [16,49]. The DNA flanking individual 'hits' was manually searched for sequence with high similarity to prototypical HML-2 sequences as determined by the RepeatMasker program in the UCSC genome browser [67]. For each identified locus, complete nucleotide sequences were generated by extracting and concatenating the internal and LTR proviral segments. Additional BLAT searches with individual K113 genes (*gag*, *pro*, *pol*, and *env*) were performed to further identify HML-2 elements within the available genome. Complete sequence reconstruction was performed as above, with the minimum criterion for a provirus being the presence of an LTR and a "hit" matching > 50% of the length of a full gene, or two proximal genes with > 50% hits and no LTR. All full-length sequences were initially aligned to K113 using ClustalW [88], and manually edited in BioEdit v.7.0.9.0 [89]. The full-length sequences for the HML-2 proviruses located at 10p12.1 (K103) and 19p12 (K113) were from NCBI (accession numbers AF164611 and AY037928, respectively). We identified the K105 sequence by taking flanking sequence of the K105 solo LTR and searching the chimpanzee database. We identified a BAC with a provirus starting at position 74813 (AC195095.2). We found a sequence with 99% similarity through a BLAST search of the NCBI database that corresponded to a human provirus labeled K111 (GU476554). Due to the high similarity between Chimpanzee K105 and this human "K111" as well as similarity between K105 deposited 5' and 3' LTRs (AH008413.1), we conclude that K111 is the human variant of the K105 provirus. Furthermore, the K111 provirus clusters most closely with chimpanzee K105 in phylogenetic trees of *gag*, *pol*, and *env*, as well as chimp and human published K105 5' and 3' LTR sequences (data not shown). The 12q13.2 provirus was sequenced in this study (described below). Provirus sequences were deposited into GenBank (accession numbers: JN675007-JN675097), along with their respective flanking sequences (accession numbers: JN675098-JN675187).

Separate searches were performed using the UCSC Genome Browser to identify chromosomal coordinates of HML-2 solo LTRs. We queried the published sequence for elements corresponding to one of three HML-2 LTR subgroups: LTR5Hs (canonical sequence is ~986 bp); LTR5A (~1004 bp); or LTR5B (~1002 bp). Sequences corresponding to solo LTRs were extracted, aligned using ClustalW, and manually edited in BioEdit v.7.0.9.0 as described above. LTRs associated, and in the same orientation, with internal HML-2 gene sequences, were excluded from this analysis to ensure that only solo LTRs were analyzed. For the remaining elements, an arbitrary cut-off of 750 bp was used to include only the most intact elements per group.

### Amplification and sequencing of the HML-2 12q13.2 provirus

The 12q13.2 solo LTR was identified in a BLAT search for K113 5'LTR related sequences in the human genome and verified by simultaneously searching the previously characterized 12q13.2-specific flanking sequence [16]. 12q13.2-specific primers were designed using Primer3 v.0.4.0 [90] for this sequence including 1 kb flanking DNA in both directions (12q13.2F: 5'-TAGGTCTAGCACACTTTATCTGTAAT-3'; 12q13.2R: 5'AGATGTCTCCATGTTAATTGC TC-3'). A panel of human DNAs [91] was screened in two PCR reactions: the first was with 12q13.2-flanking primers to detect individuals with either the solo LTR or pre-integration site; the second PCR was with the 12q13.2F primer and an HML-2-specific reverse primer (HML-2R: 5'-CTCGAGCGTACCTTCACCCTAG-3') to detect the 12q13.2 5' LTR. PCR reactions were analyzed by gel electrophoresis. Genomic DNA from one homozygous individual was selected for sequencing the full-length 12q13.2 provirus. The provirus was amplified in 4 overlapping segments using conserved primers internal to the provirus [15] paired with either 12q13.2F or 12q13.2R (PicoMaxx, Stratagene). PCR products were purified (Qiagen) and sequenced to at least 6x coverage using a previously described HML-2 primer set [15]. Individual sequence traces were manually edited and aligned with reference to the K113 nucleotide sequence in BioEdit v.7.0.9.0 [89], and the consensus sequence manually introduced into our HML-2 alignment (Additional File 1).

### Phylogenetic analyses

Within the HML-2 alignment, sequences corresponding to *gag *(position 1083-3168 with respect to the HML-2 alignment in Additional File 1), *pol *(position 5242-5899) and *env *(position 8296-9252) were extracted for phylogenetic analysis. Individual HML-2 *pol *sequences were aligned with known non-HML-2 *pol *sequences to confirm the identity of proviruses to HML-2 group. Full length sequences representing non-HML-2 HERV-Ks were retrieved using UCSC BLAT based upon the GenBank accession numbers: HML-1 (U35102, U35103, U35157, AF015999), HML-2 (U35104-U35107, U35158, AF015994), HML-3 (U35153-U35156,U35159, AF015998), HML-4 (U35160,), HML-5 (U35161, AF015995), HML-6 (U35162-U35164, AF015997), HML-7 (AF016000), HML-8 (AF015996), HML-9 (AF016001), HML-10 (U07856). Full-length MMTV (NC_001503), JSRV (M80216), and MPMV (NC_001550) genomes were also aligned to this region. Neighbor-joining trees were generated with MEGA4 using the pair-wise deletion option and 5000 bootstraps [92]. Tree topologies were confirmed using Bayesian inference (MrBayes v.3.1.2) [93,94] with four independent chains run for at least 1,000,000 generations until sufficient trees were sampled to generate > 99% credibility. LTR trees were generated using Bayesian inference as above.

### Age estimation of HML-2 proviruses and subgroups

Individual provirus ages were inferred using a neutral substitution rate of 0.24%-0.45% per million years, as previously described by Hughes and Coffin [65]. Briefly, nucleotide substitutions between cognate 5' and 3' LTRs were counted and scored as a percentage of their sequence length, with insertions or deletions of > 2 bases treated as single substitutions. The substitution frequency was divided by 0.24%/mya (for upper bound) or 0.45%/mya (for lower bound) per provirus to obtain age estimates. To estimate the relative ages of solo LTRs, they were first divided into groups based upon shared nucleotide motifs: LTR5A, LTR5B, LTR5Hs. The LTR5Hs share a 4 base insertion at the consensus position 461; LTR5A-specific insertions are found at position 135 of the LTR5A consensus; LTR5B LTRs are missing both insertions. For each group, an alignment was made using ClustalW, manually edited and used to generate group consensus sequences. Ages were estimated per group by comparing the number of substitutions between individual elements with the respective consensus sequence per LTR subgroup using the average age calculated using by the Jukes-Cantor model [68] and the Kimura 2-parameter model with κ = 2 [68]. Ages were adjusted by drawing a best-fit line using PRISM between proviral age as determined by 5' and 3' LTR comparison, and the age determined using the distance from subgroup consensus. The slope of the line going through the origin was used as an age correction factor, with slopes of lines corresponding to 95% confidence intervals being used to calculate the maximum and minimum ages. Note that since much of the divergence between LTRs at different integration sites may have occurred during virus replication prior to germline integration, ages estimated in this way are likely to be quite inaccurate.

### Statistical analyses

All statistical analyses were performed by the Data Design and Resource Center at Tufts University. Briefly, we analyzed the pair-wise relationship between proviruses, solo LTRs, RefSeq genes, and chromosome size. Values for total proviruses and solo LTRs were from the HML-2 dataset presented in this study, and values for chromosomal size and gene density were extracted respectively using the Base Position and RefSeq Genes tracks within UCSC Genome Browser. Expected frequencies for proviruses, solo LTRs, and RefSeq genes were estimated as a function of chromosome number using negative binomial regression analysis. In each model, likelihood ratio (LR) statistics were calculated: -2 (log likelihood (from the model without a predictor) - log likelihood (from the model with a predictor)) and subsequently analyzed by a χ^2 ^test with degrees of freedom equal to 1. All results had a p-value < 0.01.

## List of Abbreviations

LTR: long terminal repeat; HML: human MMTV-like; HERV: human endogenous retrovirus; BAC: bacterial artificial chromosome; mya: million years ago

## Competing interests

The authors declare that they have no competing interests.

## Authors' contributions

RPS and JHW together designed this study, performed all the extraction and analyses of HML-2 provirus sequence, as well as drafted and edited this manuscript. CR was responsible for sequencing the12q13.2 provirus and reviewing the manuscript. JMC participated in the design and coordination of the study as well as critically reviewing this manuscript. All authors have read and approved the final manuscript.

## Supplementary Material

Additional file 1 **Sequence alignment of HML-2 proviruses**. Shown are full alignments for all 89 HML-2 proviruses and 2 HML-11 proviruses. Sequences are provided as a FASTA alignment, and can be viewed as alignments in any sequence editing software (e.g. BioEdit, MEGA, MacVector, SeaView, Geneious, Mesquite, CLC Workbench), and as plain text in common word processing applications. Click here for file

Additional file 2 **HML-2 Solo LTRs in the Human Genome**. Click here for file
